# Potent *In Vitro* and *Ex Vivo* Anti-Gonococcal Activity of the RpoB Inhibitor Corallopyronin A

**DOI:** 10.1128/msphere.00362-22

**Published:** 2022-09-12

**Authors:** Jennifer L. Edwards, Jacqueline T. Balthazar, Danillo L. A. Esposito, Julio C. Ayala, Andrea Schiefer, Kenneth Pfarr, Achim Hoerauf, Silke Alt, Thomas Hesterkamp, Miriam Grosse, Marc Stadler, Daniel Golparian, Magnus Unemo, Timothy D. Read, William M. Shafer

**Affiliations:** a The Center for Microbial Pathogenesis, The Abigail Wexner Research Institute at Nationwide Children's Hospital, Columbus, Ohio, USA; b The Department of Pediatrics, The Ohio State University College of Medicine, Columbus, Ohio, USA; c Department of Microbiology and Immunology, Emory University School of Medicine, Atlanta, Georgia, USA; d Institute for Medical Microbiology, Immunology and Parasitology, University Hospital Bonn, Bonn, Germany; e German Center for Infection Research (DZIF), Partner Site Bonn-Cologne, Bonn, Germany; f Translational Project Management Office (TPMO), German Center for Infection Research, Braunschweig, Germany; g Department Microbial Drugs, Helmholtz Centre for Infection Research, Braunschweig, Germany; h German Center for Infection Research (DZIF), Partner Site Hannover-Braunschweig, Braunschweig, Germany; i WHO Collaborating Centre for Gonorrhoea and Other STIs, National Reference Laboratory for STIs, Department of Laboratory Medicine, Clinical Microbiology, Faculty of Medicine and Health, Örebro University, Örebro, Sweden; j Institute for Global Health, University College London, London, United Kingdom; k Department of Medicine, Division of Infectious Disease, Emory University School of Medicine, Atlanta, Georgia, USA; l The Emory Antibiotic Resistance Center, Emory University School of Medicine, Atlanta, Georgia, USA; m Laboratories of Bacterial Pathogenesis, Veterans Affairs Medical Center (Atlanta), Decatur, Georgia, USA; Antimicrobial Development Specialists, LLC

**Keywords:** *Neisseria gonorrhoeae*, gonorrhea, corallopyronin A, anti-gonococcal, *ex vivo* model, biofilm

## Abstract

Gonorrhea remains a major global public health problem because of the high incidence of infection (estimated 82 million cases in 2020) and the emergence and spread of Neisseria gonorrhoeae strains resistant to previous and current antibiotics used to treat infections. Given the dearth of new antibiotics that are likely to enter clinical practice in the near future, there is concern that cases of untreatable gonorrhea might emerge. In response to this crisis, the World Health Organization (WHO), in partnership with the Global Antibiotic Research and Development Partnership (GARDP), has made the search for and development of new antibiotics against N. gonorrhoeae a priority. Ideally, these antibiotics should also be active against other sexually transmitted organisms, such as Chlamydia trachomatis and/or Mycoplasma genitalium, which are often found with N. gonorrhoeae as co-infections. Corallopyronin A is a potent antimicrobial that exhibits activity against Chlamydia spp. and inhibits transcription by binding to the RpoB switch region. Accordingly, we tested the effectiveness of corallopyronin A against N. gonorrhoeae. We also examined the mutation frequency and modes of potential resistance against corallopyronin A. We report that corallopyronin A has potent antimicrobial action against antibiotic-susceptible and antibiotic-resistant N. gonorrhoeae strains and could eradicate gonococcal infection of cultured, primary human cervical epithelial cells. Critically, we found that spontaneous corallopyronin A-resistant mutants of N. gonorrhoeae are exceedingly rare (≤10^−10^) when selected at 4× the MIC. Our results support pre-clinical studies aimed at developing corallopyronin A for gonorrheal treatment regimens.

**IMPORTANCE** The high global incidence of gonorrhea, the lack of a protective vaccine, and the emergence of N. gonorrhoeae strains expressing resistance to currently used antibiotics demand that new treatment options be developed. Accordingly, we investigated whether corallopyronin A, an antibiotic which is effective against other pathogens, including C. trachomatis, which together with gonococci frequently cause co-infections in humans, could exert anti-gonococcal action *in vitro* and *ex vivo*, and potential resistance emergence. We propose that corallopyronin A be considered a potential future treatment option for gonorrhea because of its potent activity, low resistance development, and recent advances in scalable production.

## INTRODUCTION

Gonorrhea continues to be a major global public health problem, as it is the second most commonly reported bacterial sexually transmitted infection (STI) worldwide, with ca. 82 million cases estimated among adults in 2020 (1). Since the late 1930s, antibiotic therapy has been the sole approach for treating infection and reducing the spread of Neisseria gonorrhoeae in the community (2). Exacerbating the high global burden of gonorrhea is the emergence of gonococci expressing resistance to all antibiotics brought into clinical practice (2, 3). The increasing prevalence of N. gonorrhoeae strains with reduced susceptibility to azithromycin has recently prompted the replacement of ceftriaxone and azithromycin dual therapy with ceftriaxone monotherapy in the United States and some European countries (4–6).

In the absence of a protective vaccine, the continued emergence and spread of gonococcal strains expressing resistance to approved antibiotics has stimulated the development of new antibiotics to treat N. gonorrhoeae infections. In this respect, several drugs have been evaluated in pre-clinical testing. Only two (gepotidacin and zoliflodacin) (7, 8) are currently in phase 3 randomized clinical controlled trials (RCTs). Additional efforts have focused on repurposing drugs approved for other indications for their potential utility as anti-gonococcal agents (9–14). As one example, corallopyronin A inhibits the replication of some bacteria by targeting the switch region of the beta-subunit (RpoB) of the DNA-dependent RNA polymerase (RNAP) (15, 16). Corallopyronin A is shown to have activity against Gram-negative (e.g., *Wolbachia* spp. [17–20], Chlamydia spp., and *Rickettsia* spp. [21–24]) and Gram-positive (e.g., Staphylococcus aureus [25]) bacteria. Nevertheless, the development of corallopyronin A has not progressed past pre-clinical testing (17), mainly due to the non-scalable production by its natural producer, Corallococcus coralloides (26, 27). This obstacle has now been overcome by the heterologous expression of corallopyronin A in Myxococcus xanthus (26–28), resulting in >100-fold higher production volumes. We now report the potent *in vitro* and *ex vivo* activity of corallopyronin A against N. gonorrhoeae in the absence of the development of single-step, high-level corallopyronin A resistance.

## RESULTS AND DISCUSSION

### High susceptibility to corallopyronin A in gonococci.

**FIG 1:**
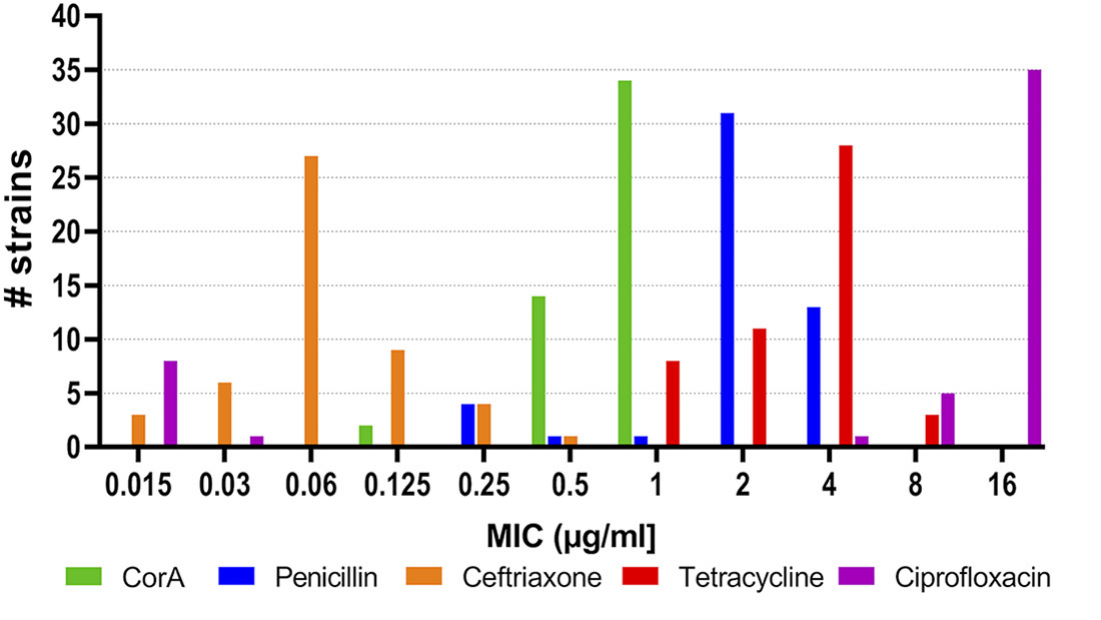
Shown are ranges of MIC values (μg/mL) against 50 Neisseria gonorrhoeae strains in a panel of antimicrobial-resistant (AMR) isolates obtained from the CDC for corallopyronin A (CorA) and antibiotics used previously or currently for the treatment of gonorrhea. The MIC values for penicillin, ceftriaxone, tetracycline, and ciprofloxacin were taken from Liu et al. (29).

To determine whether corallopyronin A can exert anti-gonococcal activity against commonly used, antibiotic-susceptible laboratory strains (FA19, FA1090, 1291, and F62), we tested a corallopyronin A dose range of 0.06 to 16 μg/mL by agar dilution. We found that CorA was active against all of these gonococcal strains, displaying a MIC range of 0.125 to 0.25 μg/mL (Table 1). To extend these findings to more recent N. gonorrhoeae strains which display resistance to previously or currently used antibiotics, we next determined the corallopyronin A MIC against a panel of 50 U.S. clinical isolates (kindly supplied by the U.S. Centers for Disease Control and Prevention [CDC]) that express different antibiotic resistance phenotypes; the antibiotic resistance properties of these strains have been published (29). As shown in Fig. 1, the corallopyronin A MIC against this panel of strains ranged from 0.125 to 1.0 μg/mL, with most showing MICs of 0.5 μg/mL (14/50) or 1.0 μg/mL (34/50). We also determined the corallopyronin A MIC against the 2016 WHO gonococcal reference strains, which have shared or distinct antibiotic resistance phenotypes (30) and include strains with resistance to previously (ciprofloxacin, penicillin, and tetracycline) or currently used (ceftriaxone and azithromycin) antibiotics. The examined international isolates (WHO M, WHO X, WHO Y, and WHO Z) were 4-fold less susceptible to corallopyronin A than the antibiotic-susceptible strains, such as FA19, with an MIC of 0.5 μg/mL (Table 1).

**TABLE 1:** Anti-gonococcal activity of CorA

Strain	CorA MIC (μg/mL)^*a*^	Source
F62	0.25	S. Morse
FA1090	0.125	M. Hobbs
1291	0.125	52
FA19	0.125	P.F. Sparling
FA19 *mtrR27*	0.5	Transformant; this study
WHO M	0.5	30
WHO X	0.5	30
WHO Y	0.5	30
WHO Z	0.5	30

a CorA: corallopyronin A. MIC values are representative from at least three independent tests.

### Loss of the MtrCDE multidrug efflux pump increases gonococcal susceptibility to corallopyronin A.

Gram-negative enteric bacteria, such as Escherichia coli, are reported to be intrinsically resistant to corallopyronin A. However, the loss of TolC in E. coli can result in corallopyronin A susceptibility (25). TolC is used by multiple drug efflux pumps in the Resistance-Nodulation-Division (RND) family as the outer membrane protein component of the tripartite pump machinery. To determine whether any of the five gonococcal efflux pumps (possessed by all N. gonorrhoeae strains [31]) can recognize corallopyronin A as a substrate for export, we tested the corallopyronin A susceptibility of wild-type (WT) strain FA19 and a corresponding panel of previously constructed genetic knockout (KO) transformants, each of which lack a single efflux pump (32). As shown in Table S1, only the loss of the MtrCDE efflux pump resulted in an increased (4-fold) susceptibility of N. gonorrhoeae to corallopyronin A. The MtrCDE efflux pump is known to export a multitude of widely diverse antimicrobials and is a member of the RND family of efflux pumps, with MtrE being structurally and functionally similar to TolC (31).

10.1128/msphere.00270-22.2 TABLE S1 MICs for FA19 N. gonorrhoeae strains. Download Table S1, DOCX file, 0.01 MB. Copyright © 2022 Edwards et al. 2022 Edwards et al. https://creativecommons.org/licenses/by/4.0/ This content is distributed under the terms of the Creative Commons Attribution 4.0 International license.

### Low frequency of gonococcal corallopyronin A resistance selected *in vitro*.

To determine whether N. gonorrhoeae can develop high-frequency, spontaneous resistance to corallopyronin A (15), we selected for spontaneous mutants of strain FA19 at 2× and 4× the MIC (0.25 and 0.5 μg/mL, respectively). In three different experiments, and at 2× the MIC, the mean spontaneous mutation frequency to decreased corallopyronin A susceptibility was 7 × 10^−8^. In contrast, at 4× MIC, no colonies were recovered. Thus, the estimated frequency of corallopyronin A resistance at this level of selection was ≤10^−10^, which is 3 orders of magnitude lower than that observed by others for S. aureus when the same degree of selection was applied (17). Based on these results with the highly corallopyronin A-susceptible strain FA19, we next examined whether more recent, multidrug-resistant (MDR) N. gonorrhoeae isolates (WHO Y and WHO X) are intrinsically less susceptible to corallopyronin A, and thereby might have a higher frequency of spontaneous resistance mutations and/or readily develop higher corallopyronin A MICs. Using a selection of 4× the corallopyronin A MIC, in four different experiments, we were unable to recover colonies from selective agar plates (frequency ≤ 10^−10^) for both strains. Thus, we conclude that, unlike other bacteria (15–17), N. gonorrhoeae does not easily develop a level of corallopyronin A resistance in the presence of ≥4× MIC. However, as described below, it is possible at 2× MIC to recover spontaneous mutants of the highly corallopyronin A-susceptible strain FA19 that are capable of expressing reduced (4-fold) susceptibility to corallopyronin A compared to their parent.

As a control, we selected for spontaneous mutants of strain KH15 (see below) capable of growth on gonococcal base (GCB) agar + 4× the MIC (MIC = 0.125 μg/mL) of rifampicin, which, like corallopyronin A, targets RpoB. We readily (frequency = 3 × 10^−8^) isolated spontaneous mutants with increased MICs in which the rifampicin MIC increased from 0.125 μg/mL to 0.5 to 2 μg/mL in three different mutants. However, these mutants did not show increased MICs for corallopyronin A (data not shown). Indeed, DNA sequencing of *rpoB* PCR products from these mutants showed that, compared to the parental strain, they had single amino acid changes in RpoB at positions 561 (Gly to Ser; MIC = 2.0 μg/mL), 597 (Gly to Ser; MIC = 0.5), or 601 (Ser to Leu; MIC = 0.5 μg/mL). Notably, high-level rifampicin resistance is linked to a single amino acid change in RpoB (His to Asn at residue 553) (33), and this mutation is present in strain WHO M (30), which expresses such resistance. However, the corallopyronin A MIC for WHO M was identical to that observed for the other WHO reference strains which possess a WT *rpoB* (Table 1). Thus, we conclude that, whereas N. gonorrhoeae can tolerate RpoB mutations resulting in rifampicin resistance, mutations in the corallopyronin A target switch region occur less easily; this may explain why it was not possible, with three different strains, to isolate spontaneous mutants expressing at least a 4-fold increase in the respective corallopyronin A MIC.

### Increased expression of the MtrCDE efflux pump can decrease gonococcal corallopyronin A susceptibility.

FA19 colonies that survived selection at 2× the corallopyronin A MIC were passed onto GCB agar containing 0.25 μg/mL corallopyronin A, and one surviving colony was selected for antimicrobial susceptibility testing and DNA sequencing studies. This selected spontaneous mutant showed a 4-fold increase in the corallopyronin A MIC compared to the FA19 parental strain. To determine whether the spontaneous mutant had a mutation in *rpoB*, we PCR-amplified this gene from the FA19 mutant and parent strains for DNA sequencing. Importantly, the *rpoB* sequences from each strain were identical (data not shown), suggesting that the decreased corallopyronin A susceptibility observed for the mutant was independent of *rpoB* and located elsewhere on the N. gonorrhoeae chromosome.

To detect mutations that might be linked to the modest decrease in corallopyronin A susceptibility observed in the mutant, we performed whole-genome sequencing (WGS) on the parent and mutant strains. The whole-genome sequencing further verified the lack of any *rpoB* mutations. However, a missense mutation at codon 27 in the *mtrR* coding sequence was identified. This mutation would cause an amino acid change (glycine [G] to arginine [R]) in the mutant MtrR protein. The selected mutant, with decreased corallopyronin A susceptibility, did not contain any other mutations compared to the parental strain. MtrR is a transcriptional repressor of the downstream *mtrCDE* efflux pump operon (34, 35). Although G27 in the WT MtrR protein is positioned adjacent to the DNA-binding domain (amino acids 33 to 52), we hypothesized that the introduction of a positive charge by arginine at position 27 (R27) in the mutant could relieve MtrR repression of *mtrCDE*, resulting in elevated expression of the MtrCDE efflux pump. Accordingly, we transformed strain FA19 with an *mtrR* PCR product from the corallopyronin A (G27R) mutant described above. Transformants were selected on GCB agar containing 0.5 μg/mL erythromycin. Three randomly picked transformants were screened and all were found to be 4-fold less susceptible to corallopyronin A than the FA19 parental strain; the corallopyronin A MIC against a representative transformant (FA19 *mtrR27*) was 0.5 μg/mL (Table 1). Moreover, the transformants were also 4-fold less susceptible to azithromycin (MIC of parental strain = 0.125 μg/mL compared to 0.5 μg/mL for transformant strains), which is a known substrate of the MtrCDE efflux pump (31, 33). Importantly, DNA sequencing of the *mtrR* coding region in the transformants confirmed the presence of the missense mutation at codon 27 and no other mutations.

**FIG 2:**
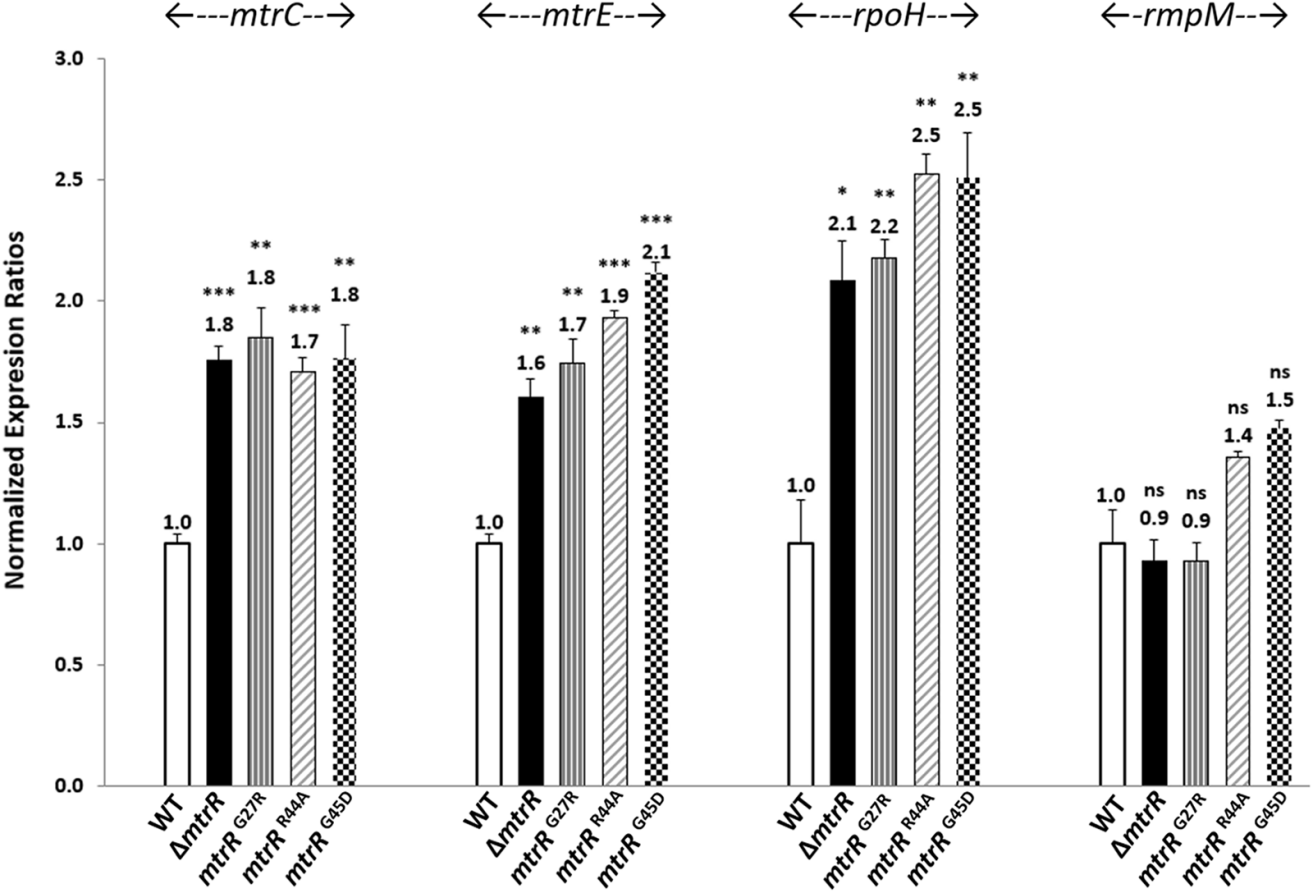
Effect of *mtrR* mutations on the expression of MtrR-regulated genes. N. gonorrhoeae wild-type strain FA19 and isogenic mutants bearing *mtrR* deletion or missense mutations G27R, R44A, and G45D were grown in gonococcal base (GC) broth to late-exponential phase before total RNA extraction. Data are depicted as the mean + standard error of the mean (error bar) of at least 3 biological and 3 technical samples. Gene expression was normalized to wild-type levels using *recA* mRNA as an internal reference gene, as described in Materials and Methods. Statistical differences were determined using a two-tailed *t* test (GraphPad Prism v5.0) with the wild type. *, *P* < 0.05; **, *P* < 0.01; ***, *P* < 0.001; ns, non-significant.

To test our hypothesis that the G27R change in MtrR would alter expression of *mtrCDE*, we used reverse transcriptase quantitative PCR (qRT-PCR) to examine the expression levels of known MtrR-regulated genes (*mtrC*, *mtrE* and *rpoH*) (36, 37) as well as a control gene (*rmpM*). Critically, the impact on gene transcription of the missense mutation at position 27 was similar (Fig. 2) to that of other FA19 transformants from previous studies which have a deleted *mtrR* coding region or missense mutations in the DNA-binding domain (R44A or G45D) and have been previously shown to reduce MtrR-binding to the *mtrC* or *rpoH* promoter regions (37). Given the ability of the MtrR G27R mutant to reduce the repressive activity on *mtrC* and *mtrE* (the last gene in the *mtrCDE* operon), and that the associated MtrCDE efflux pump is needed for the WT corallopyronin A MIC phenotype, we asked how frequently the mutation might occur in the N. gonorrhoeae population represented by public genome sequence data. To this end, we created a database of proteins from 731 gonococcal genomes (based on NCBI taxonomic ID of 485, see Materials and Methods). From this database, we identified 35 unique MtrR alleles, none of which had the G27R substitution. Thus, the missense mutation identified in the spontaneous FA19 mutant in this study is rarely, if ever, found in existing N. gonorrhoeae isolates. Further, analysis of 32,375 publicly available gonococcal genomes showed that none would encode the G27R mutant MtrR protein (data not presented).

Increased expression of the *mtrCDE* operon due to the G27R amino acid substitution in MtrR resulted in decreased corallopyronin A susceptibility; therefore, we asked whether a common *cis*-acting, single-bp deletion in the *mtrR* promoter, which is known to elevate *mtrCDE* expression and antimicrobial resistance above *mtrR* coding mutations (38, 39), could similarly decrease N. gonorrhoeae corallopyronin A susceptibility. For this purpose, we used strain FA19 and its isogenic transformant KH15 (38), which has the *mtrR* promoter mutation, and found that the latter was 8-fold less susceptible to corallopyronin A than the FA19 parent strain (1.0 versus 0.125 μg/mL, respectively). Examination of the corallopyronin A susceptibility of the N. gonorrhoeae strain FA6140, which also has the *cis*-acting, single bp deletion in the *mtrR* promoter and caused a penicillin-resistant gonorrhea outbreak in the US in 1983 (40), showed a corallopyronin A MIC of 1.0 μg/mL. Lastly, we noted that 39/50 N. gonorrhoeae strains in the CDC Antimicrobial Resistance Isolate (AMR) Bank were reported to have this single-bp deletion in the *mtrR* promoter (28), but in all cases the corallopyronin A MIC was ≤1 μg/mL (Fig. 1).

### Corallopyronin A is effective in curing *ex vivo N. gonorrhoeae* infection of human cervical cells.

**FIG 3:**
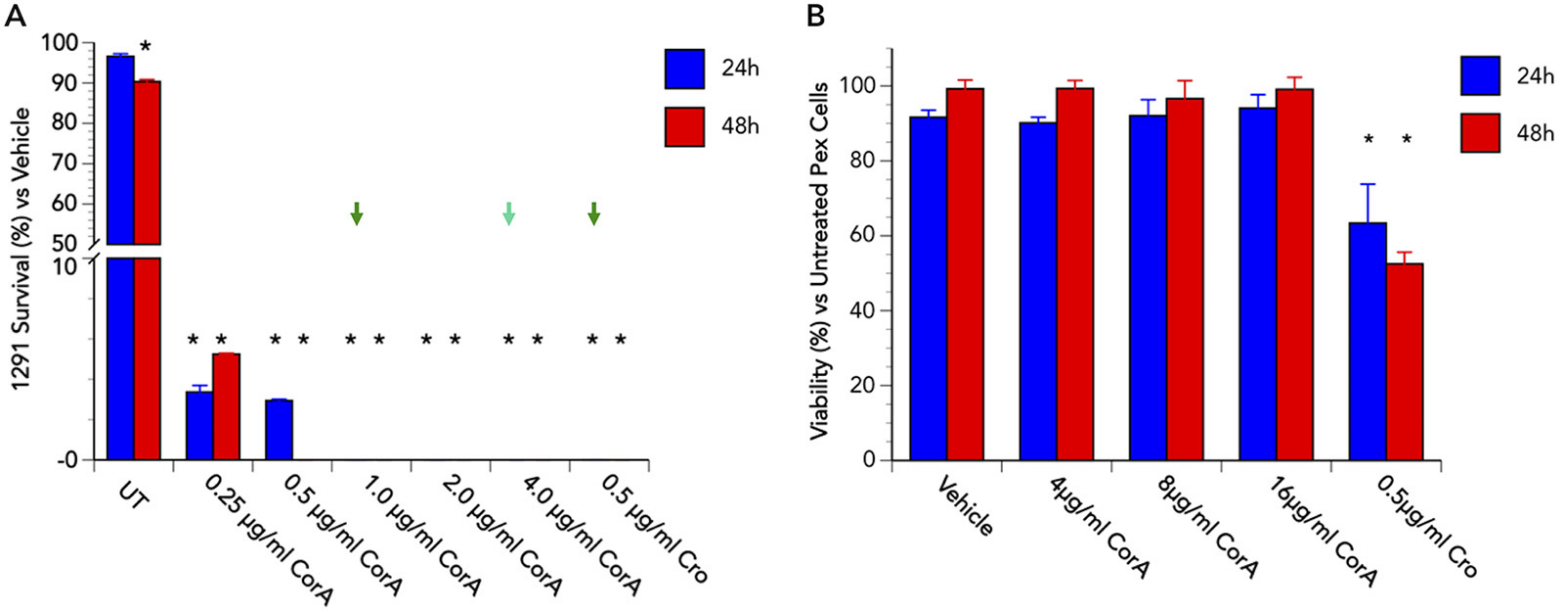
Single-dose corallopyronin A treatment is effective against N. gonorrhoeae strain 1291 and is not cytotoxic to Pex cells. (A) The ability of strain 1291 to survive a dose range (*x* axis) of corallopyronin A (CorA) treatment during Pex cell infection was examined as described in the text. Values shown are the means (variance) of the percentage of viable gonococci recovered at 24 or 48 h post-CorA or ceftriaxone (Cro) treatment or from untreated (UT) Pex cell infections, compared to the number of viable bacteria recovered following treatment with the dimethyl sulfoxide (DMSO) vehicle control at the same time point. Data were obtained from three trials performed in triplicate. Arrows indicate the lowest dose of CorA (or Cro) in which no viable gonococci were recovered from Pex cell lysates at 24 h (light green, shortest duration of effective treatment) or 48 h (dark green, lowest dose required for effective treatment irrespective of duration) post-treatment. *, *P ≤ *0.0001 versus vehicle control. (B) Potential CorA cytotoxicity toward uninfected Pex cells was examined at 24 and 48 h post-incubation of Pex cells treated with 0.1% DMSO (vehicle), Cor A (4, 8, or 16 μg/mL), or 0.5 μg/mL Cro, or of untreated cells, as described in the text. Data are shown as the mean (variance) percentage of fluorescence recorded for each condition as a function of fluorescence recorded for untreated Pex cells. Assays were performed in duplicate on 3 separate occasions, and a Student’s *t* test was used to determine the statistical significance of data obtained. CorA had no significant (*P* ≥ 0.4986) effect on Pex cell viability. However, treatment with 0.5 μg/mL Cro surprisingly resulted in moderately decreased Pex cell viability. *, *P ≤ *0.0033 versus untreated cells.

**FIG 4:**
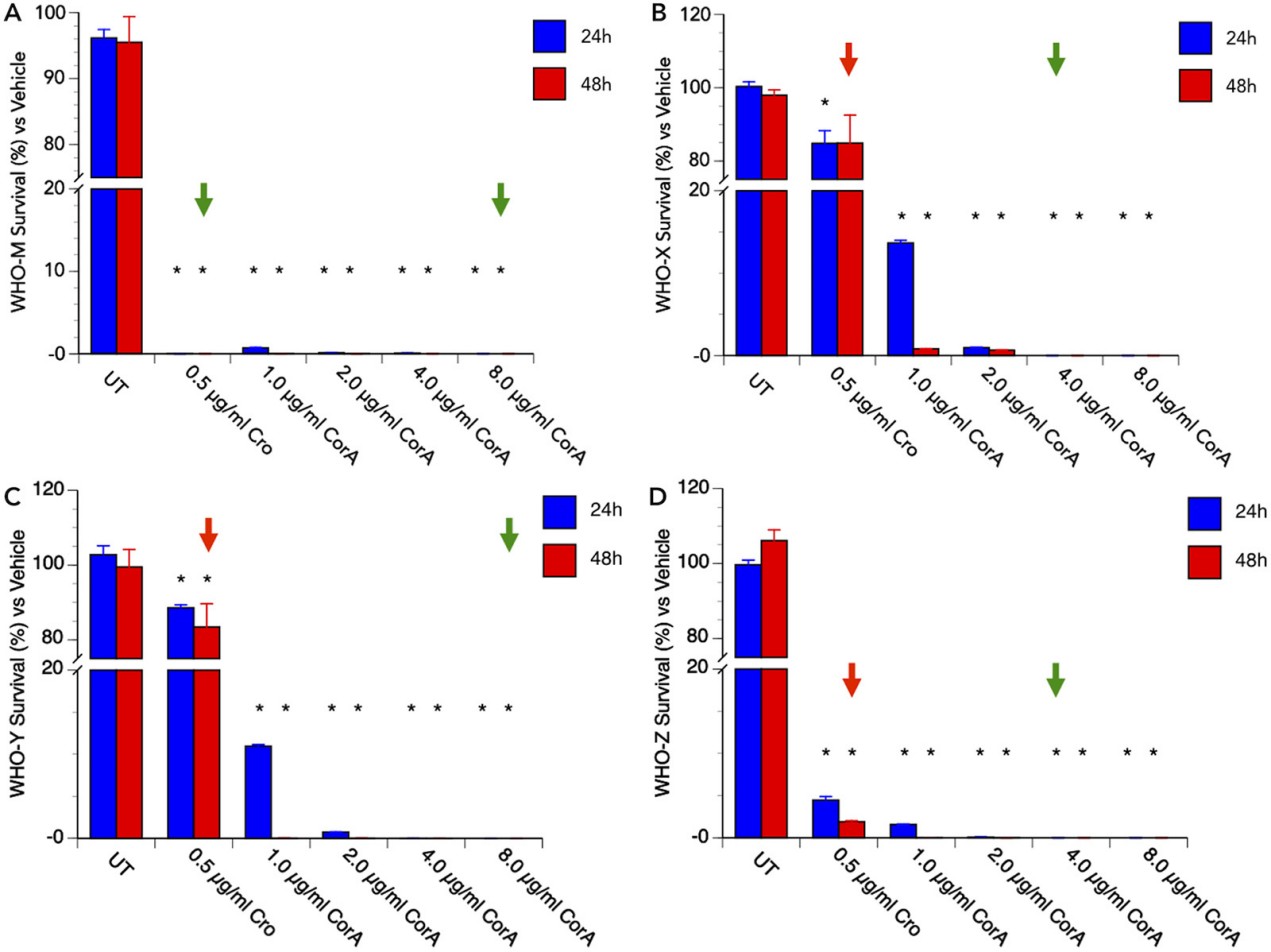
Corallopyronin A is effective in curing cervical cell infections with multidrug-resistant (MDR) or extensively drug-resistant (XDR) N. gonorrhoeae. The ability of strains (A) WHO M, (B) WHO X, (C) WHO Y, and (D) WHO Z to survive a dose range (*x* axis) of corallopyronin A (CorA) treatments during Pex cell infection was examined, as described in the text. Values shown are the means (variance) of the percentage of viable gonococci recovered at 24h (blue bars) or 48 h (red bars) from untreated (UT) Pex cell infections or following CorA or ceftriaxone (Cro) treatment, compared to the number of viable bacteria recovered following treatment with the DMSO (vehicle) control at the same time point. Data were obtained from three trials performed in triplicate. (A to D) Green arrows indicate the lowest dose and shortest duration of effective treatment (no viable gonococci recovered from Pex cell lysates) for each strain post-CorA (or Cro) treatment. (B to D) Red arrow indicates that a 48-h, 0.5-μg/mL treatment of Cro was not effective in “curing” Pex cell infection. *, *P ≤ *0.0196 versus vehicle.

As described above, corallopyronin A was effective against N. gonorrhoeae in an *in vitro* culture with an MIC of ≤ 1 μg/mL. To assess the ability of corallopyronin A to cure an established N. gonorrhoeae infection in a biological system, we performed “cure” assays under physiological conditions (3% O_2_) using primary human cervical epithelial (i.e., Pex) cells. In brief, Pex cells were challenged with N. gonorrhoeae for 90 min and then either left untreated or treated with dimethyl sulfoxide (DMSO; vehicle control), corallopyronin A, or ceftriaxone for 24 or 48 h. Initial experiments were performed using N. gonorrhoeae 1291, a laboratory strain that has been maintained at a low-passage-number and displays high susceptibility to corallopyronin A (Table 1). Compared to untreated or vehicle-treated infections, a single dose (1 μg/mL), 24-h treatment with corallopyronin A resulted in a >99.99% reduction in the number of viable N. gonorrhoeae recovered from Pex cells, and no viable bacteria were recovered following a 48-h treatment with a single ≥0.5-μg/mL dose of corallopyronin A (Fig. 3A). Moreover, no cytotoxicity was observed when uninfected Pex cells were incubated with corallopyronin A at concentrations of up to 16 μg/mL for 48 h (Fig. 3B). A >99% reduction in viable N. gonorrhoeae recovered from Pex cells was also observed when we repeated these assays using the multidrug-resistant or extensively drug-resistant (XDR) strains WHO M, WHO X, WHO Y, and WHO Z which, as a group, are intrinsically less susceptible than 1291 to corallopyronin A (Table 1). However, these infections were not completely eradicated by 48-h treatment with corallopyronin A at 2 μg/mL (Fig. S1), which is 4× its MIC against these isolates. We, therefore, extended the dose range (0 to 8 μg/mL) to again test the effectiveness of corallopyronin A against these same MDR or XDR strains and found that, for all strains tested, 100% bacterial killing was observed by 24 h with a single, ≤8-μg/mL dose of corallopyronin A (Fig. 4). In contrast, although ceftriaxone (0.5 μg/mL) was effective at clearing WHO M infection of Pex cells, it was ineffective in resolving Pex cell infections with WHO X, WHO Y, or WHO Z. Thus, these data support the future development and use of corallopyronin A to treat MDR and XDR N. gonorrhoeae infections of the cervix.

10.1128/msphere.00270-22.2 FIG S1 Effect of low dose corallopyronin A on MDR and XDR gonococci. Download FIG S1, DOCX file, 0.13 MB. Copyright © 2022 Edwards et al. 2022 Edwards et al. https://creativecommons.org/licenses/by/4.0/ This content is distributed under the terms of the Creative Commons Attribution 4.0 International license.

### Corallopyronin A is effective against *N. gonorrhoeae* biofilms.

Gonococcal cervicitis is associated with the development of a biofilm (41, 42). Therefore, we examined the ability of corallopyronin A to prevent biofilm formation as well as to disrupt an established biofilm using the MDR or XDR strains noted above, both in the Pex cell model and on an abiotic surface. To our knowledge, the ability of strains WHO M, WHO X, WHO Y, and WHO Z to form a biofilm has not been previously evaluated. Therefore, we first performed scanning electron microscopy (SEM) to determine whether each of these strains was capable of biofilm formation on Pex cells. A biofilm was readily visible on the Pex cell surface by day 4 postinfection with each of the strains tested (data not shown).

**FIG 5:**
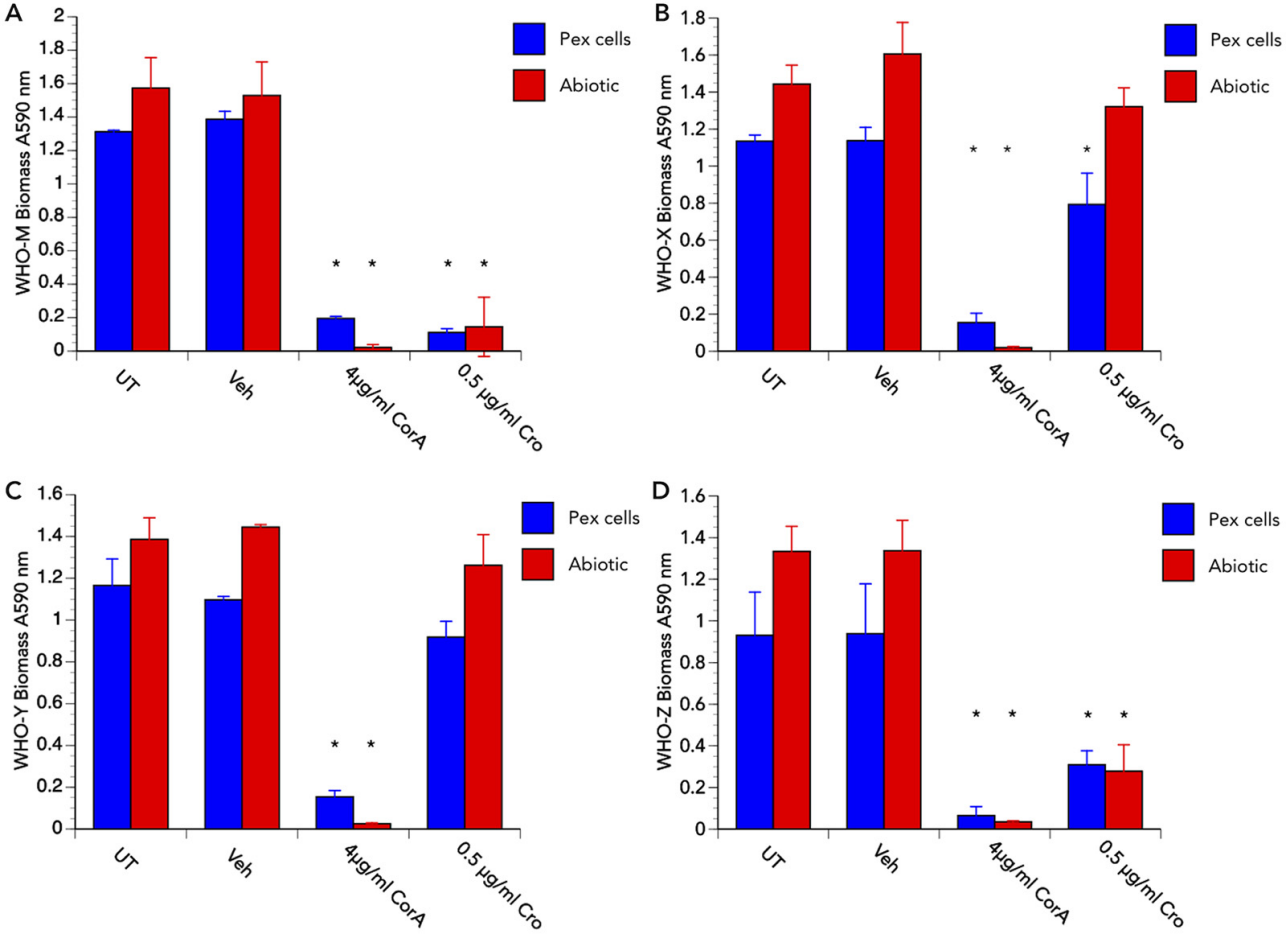
Corallopyronin A is effective in preventing N. gonorrhoeae biofilm formation. The ability of corallopyronin A (CorA) to prevent biofilm formation by strains (A) WHO M, (B) WHO X, (C) WHO Y, and (D) WHO Z on Pex cells (blue bars) and abiotic plastic tissue culture dishes (red bars) was assessed by crystal violet retention, indicative of biomass, as described in the text, at 4 days post-infection/inoculation. Data were adjusted for background and/or for biomass recorded for uninfected Pex cells; thereby, data presented are the biofilm biomass recorded only for each strain. Data shown represent the means (variance) from triplicate wells from 3 experiments performed on separate occasions. UT, untreated, Veh, DMSO vehicle control. *, *P ≤ *0.0267 versus vehicle control.

**FIG 6:**
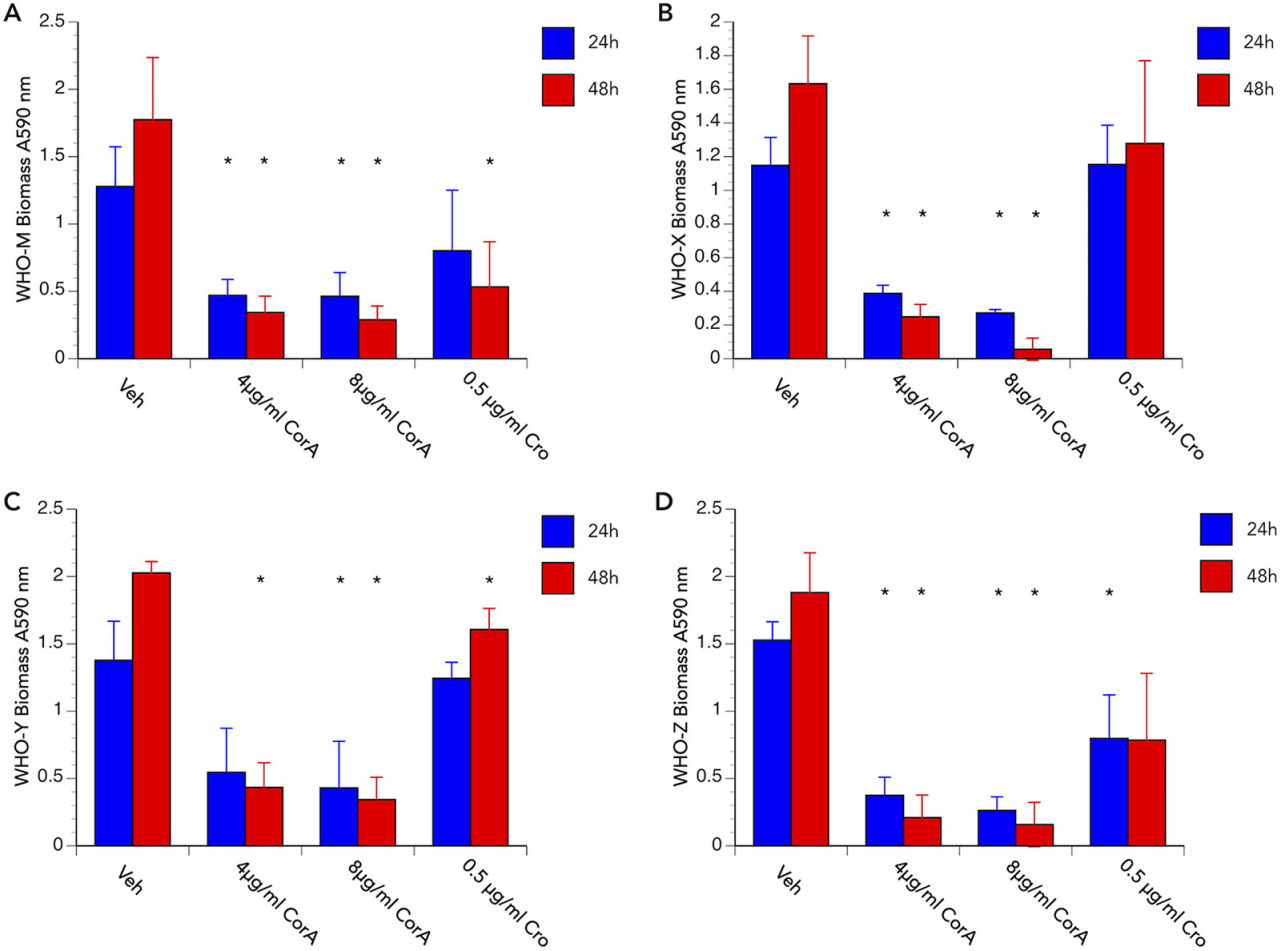
Corallopyronin A is effective in disrupting an established N. gonorrhoeae biofilm on Pex cells. The ability of corallopyronin A (CorA) to disrupt a 4-day biofilm, formed with Pex cell infection with strains WHO M (A), WHO X (B), WHO Y (C), and WHO Z (D), was determined following a 24-h (blue bars) or 48-h (red bars) treatment with CorA or ceftriaxone (Cro), as noted on the *x* axis. Biofilm biomass was assessed by crystal violet retention, as described in the text. Data were adjusted for background and for biomass recorded for uninfected Pex cells; thereby, data presented are the biofilm biomass recorded only for each strain. Data shown represent the means (variance) from triplicate wells from 3 experiments performed on separate occasions. *, *P ≤ *0.0412 versus vehicle control.

We next set out to determine the potential utility of corallopyronin A for preventing and treating N. gonorrhoeae biofilms by recording crystal violet (CV) retention as a measurement of biofilm biomass. As expected, based on data obtained from treatment assays (Fig. 3 and 4, Fig. S1), a 24-h treatment with 4 μg/mL corallopyronin A dramatically impaired the ability of all N. gonorrhoeae strains tested to form a biofilm on Pex cells or on plastic tissue culture dishes (Fig. 5). To determine whether corallopyronin A could disrupt an established biofilm, biofilms were allowed to form for 4 days on Pex cells or plastic, after which they were treated with vehicle control (0.1% [vol/vol] DMSO), corallopyronin A (4 or 8 μg/mL), or ceftriaxone (0.5 μg/mL) for 24 or 48 h before the measurement of biofilm biomass by CV retention. For all N. gonorrhoeae strains tested, biofilm biomass on Pex cells was significantly (*P ≤ *0.0412) reduced following a single dose, 24-h treatment with corallopyronin A (Fig. 6). Biofilm biomass was further reduced by a 48-h, single-dose treatment with corallopyronin A, and it was more effective than ceftriaxone in disrupting an established biofilm (Fig. 6). The reduced ability (versus corallopyronin A) of ceftriaxone to disrupt an established N. gonorrhoeae biofilm on Pex cells was not totally dependent upon the ceftriaxone-susceptibility phenotype for each strain tested. That is, biofilm biomass was reduced only ~30% for the ceftriaxone-susceptible strain WHO M, whereas a 58% reduction in biomass was observed following ceftriaxone treatment of biofilms formed by strain WHO Z, which exhibits intermediate ceftriaxone resistance. Similar data were observed when corallopyronin A was used to disrupt N. gonorrhoeae biofilms on an abiotic surface (Fig. S2).

**FIG 7:**
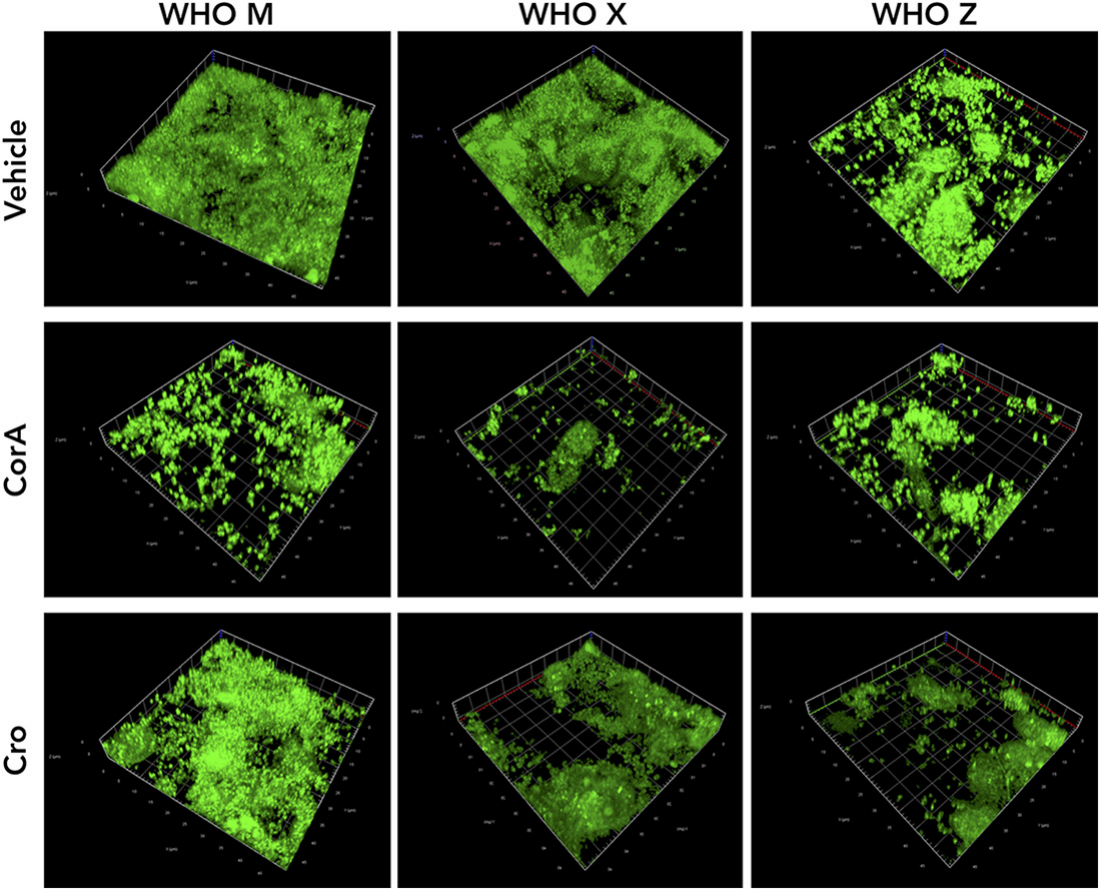
Three-dimensional composite image of the effect of corallopyronin A on established N. gonorrhoeae biofilms on Pex cells. The ability of corallopyronin A (CorA) to disrupt a 2-day biofilm, formed with Pex cell infection with strains WHO M, WHO X, or WHO Z, was visualized by confocal microscopy following a 48-h treatment with 0.1% DMSO (vehicle control), 8 μg/mL CorA, or 0.5 μg/mL ceftriaxone (Cro). Biofilms were stained using Syto-9 (green channel) and propidium iodide (red channel) (i.e., Live/Dead BacLight Bacterial Viability kit), as described in the text. To avoid potentially confounding fluorescence resulting from Pex cell staining, 3D images were constructed from 1-μm Z-sliced images of bacteria above the plane of Pex cells; therefore, only N. gonorrhoeae are visible in the representative images. Dead bacteria (red channel) are not readily visible as a result of washing the biofilms before the staining procedure. Magnification is ×63 with oil emersion.

As an additional approach, we used confocal microscopy to examine the effect of corallopyronin A on N. gonorrhoeae biofilms. Biofilms were allowed to form for 2 days on Pex cells, or on an abiotic surface, before a 48-h treatment with DMSO, corallopyronin A, or ceftriaxone. Biofilms were then stained using the BacLight Bacterial Viability kit to allow visualization and/or the quantification of biofilm biomass and bacterial viability. Qualitative assessment of 3D images of N. gonorrhoeae biofilms on Pex cells revealed that the biofilms were diminished with corallopyronin A treatment (Fig. 7). Although dead bacteria were not readily visible in biofilms on Pex cells, this was most likely the result of washing the biofilms before the staining procedure. Indeed, dead bacteria were readily visible upon confocal microscopic analyses of biofilms formed on plastic chamber slides in the absence of Pex cells, which were not rinsed before staining to preserve the biofilm structure. This observation was most pronounced for corallopyronin A-treated biofilms (Fig. S3A), which was confirmed by Comstat analyses of N. gonorrhoeae viability as a function of total biomass in abiotic, chamber slide biofilms (Fig. S3B). Collectively, our data indicate that corallopyronin A may have utility in treating N. gonorrhoeae biofilms, which are observed during *in vivo* cervical infection.

10.1128/msphere.00270-22.2 FIG S2 Abiotic biofilm disruption. Download FIG S2, DOCX file, 0.26 MB. Copyright © 2022 Edwards et al. 2022 Edwards et al. https://creativecommons.org/licenses/by/4.0/ This content is distributed under the terms of the Creative Commons Attribution 4.0 International license.

10.1128/msphere.00270-22.2 FIG S3 Confocal microsopic analyses of abiotic biofilms. Download FIG S3, PDF file, 0.31 MB. Copyright © 2022 Edwards et al. 2022 Edwards et al. https://creativecommons.org/licenses/by/4.0/ This content is distributed under the terms of the Creative Commons Attribution 4.0 International license.

### Conclusion.

Our finding that corallopyronin A displays potent anti-gonococcal action against multiple N. gonorrhoeae isolates without detectable emergence of spontaneous resistance at 4× the corallopyronin A MIC provide a foundation for continued research on this RpoB inhibitor as a future antibiotic to treat gonorrheal infections. Thus, recent advances in the scalable production of corallopyronin A (28), the development and use of the hollow fiber infection model to study the pharmacodynamics of antibiotics active against N. gonorrhoeae (43), and the development of a murine model to examine the *in vivo* efficacy of antibiotics during gonococcal infection of the female genital tract (44) should collectively facilitate such work.

One important finding of this work is the difficulty N. gonorrhoeae had in developing corallopyronin A resistance in a single step, as has been described for S. aureus (16, 45). It is noteworthy that, in the antibiotic-susceptible strain FA19, the selection of increased MICs (4-fold) of corallopyronin A at 2× MIC resulted in the isolation of a spontaneous mutant displaying a 4-fold decrease in corallopyronin A susceptibility. This mutant had a single amino acid change in the MtrR transcriptional repressor that, in WT strains, dampens expression of the *mtrCDE* efflux pump operon (35–37). This observation, coupled with the finding that loss of the MtrCDE efflux pump increases gonococcal susceptibility to corallopyronin A, suggests that the active export of corallopyronin A through the MtrCDE efflux pump is an intrinsic mechanism by which gonococci can reduce the anti-gonococcal action of corallopyronin A.

Gonococcal cervicitis is associated with the formation of a N. gonorrhoeae biofilm (42), and bacterial biofilms are notorious for being intractable to antibiotic treatment. Thereby, our finding that corallopyronin A was effective in both preventing and disrupting an established N. gonorrhoeae biofilm underscores the importance of the further development of corallopyronin A as a potential therapy or prophylactic for gonococcal cervicitis and, potentially, for infection at other anatomical sites. Although corallopyronin A treatment was not 100% effective at disrupting established N. gonorrhoeae biofilms on Pex cells, biofilm biomass was reduced more than 83% following a 48-h, single-dose treatment with 8 μg/mL corallopyronin A. This represents a significant improvement over ceftriaxone, which reduced biofilm biomass only by ca. 30% for the ceftriaxone-susceptible strain WHO M. Whether the effectiveness of corallopyronin A against gonococcal biofilms can be further augmented by co-treatment with other molecules known to target the biofilm matrix (e.g., nucleases or DNA-binding proteins) is an avenue for future study. Similarly, it may be of interest to determine the potential effects of external stressors, such as corallopyronin A, on the expression of gonococcal *nuc*, which is shown to digest DNA within the biofilm matrix (46).

The future, with regard to effective anti-gonococcal therapy, is uncertain as MDR and XDR strains will most likely continue to emerge, and there is a paucity of potential new anti-gonococcal therapies in the pipeline. The data described herein (Fig. 3B), as well as those described by others (47), indicate that corallopyronin A is not cytotoxic at doses we have shown to be therapeutically effective against N. gonorrhoeae. Additionally, corallopyronin A is shown to be effective against Chlamydia trachomatis infection *ex vivo*, in a human fallopian tube organ culture model, as well as *in vivo*, in a mouse model of genital tract infection (22). These properties, together with the apparent limited ability of gonococci to develop resistance mutations, support the continued development of corallopyronin A as an anti-gonococcal agent.

## MATERIALS AND METHODS

### *N. gonorrhoeae* strains and growth conditions.

The list of N. gonorrhoeae strains employed in this study are provided in Table 1 and Table S1. Gonococci were grown on GCB agar (Difco, Sparks, MD) containing Kellogg’s supplement I and modified supplement II (48) at 37°C under 5.0% (vol/vol) CO_2_-enriched humid atmosphere (34). Alternatively, for Pex cell infection studies, N. gonorrhoeae were grown on GCB agar in a mixed gas incubator under microaerobic conditions (3% O_2_) to simulate the *in vivo* cervical environment. Liquid cultures of gonococci for growth assays were begun by inoculating plate-grown cells in pre-warmed GCB broth containing Kellogg’s supplements I and II (modified) and 0.043% (wt/vol) sodium bicarbonate and grown in a 37°C water bath with shaking.

### Production and purification of corallopyronin A.

High quality, research-grade corallopyronin A was produced at the Department of Microbial Drugs, Helmholtz Centre for Infection Research (Braunschweig, Germany), using the heterologous producer strain Myxococcus xanthus *DK1622*::*pDPO-mxn116-Pvan-Tpase*, as previously described (27, 28). In brief, the heterologous host was maintained in 50% glycerol as cryo-cultures at −80°C. Agar plates of CTT medium supplemented with kanamycin were inoculated with 500 μL of the working cell bank. After incubation at 30°C for 72 h, cells were harvested from the agar surface by scraping and used to inoculate the first liquid seed culture in M7/S4 medium and incubated at 30°C for 48 h with 180 rpm in an orbital shaker (Multitron Pro, Infors HT AG, Basel, Switzerland). This material was used to inoculate a second seed culture comprised of 1% grown cells in fresh M7/S4 medium that was incubated for 48 h as previously. From the second seed culture, 10% was transferred to the seed 15-L stirred-tank bioreactor (C10-3, BBI-Biontech GmbH, Berlin, Germany) containing M7/S4 medium without HEPES (10% inoculation density). The set points are as follows: temperature, 30°C; pH 7.4 ± 0.1; aeration, 0.005 volume of air per volume of liquid per minute (vvm). Twenty percent partial O_2_ pressure was maintained by increasing the stirring speed using three Rushton impellers. The production process was performed in a 150-L stirred-tank bioreactor (Proreact Heinrich Frings GmbH and Co. KG, Rheinbach, Germany) containing M7/S6 medium supplemented with vanillic acid. Process parameters were maintained as in the seed bioreactors. After cultivating for 144 h, the culture supernatant was stirred with Amberlite XAD-16 resin for the extraction of corallopyronin A. The resin was recovered by sieving and purged with buffered 50% aqueous methanol. Enriched corallopyronin A was eluted with buffered methanol. The methanol was evaporated and raw corallopyronin A was provided by ethyl acetate extraction of the diluted remaining buffer. Pure corallopyronin A (>90%) was achieved by reversed phase chromatography on a medium-pressure liquid chromatography (MPLC) column YMC OSD-AQ (12 nm, 20 μm; YMC Europe GmbH, Dinslaken, Germany) with 30% buffered aqueous acetonitrile. Stock corallopyronin A solutions were prepared by dissolving in 100% DMSO and stored at −80°C until used.

### MIC determination.

The MICs of antimicrobials, including corallopyronin A, were determined in the laboratory of W.M.S. by the agar dilution method essentially as described by the Clinical and Laboratory Standards Institute (49) and used in the past for N. gonorrhoeae (34, 48, 49), but without hemoglobin added to the GCB agar. The inoculum consisted of 5 μL containing approximately 5 × 10^5^ CFU, which was obtained from overnight GCB agar-grown cultures as described above and resuspended in GCB broth to give approximately 10^8^ CFU/mL. Antimicrobials other than corallopyronin A were purchased from Sigma Chemical Co. (St. Louis, MO). Plates were incubated for 24 h at 37°C under a 5.0% (vol/vol) CO_2_-enriched humid atmosphere before being photographed for data storage purposes. We considered ≥4-fold differences in corallopyronin A or rifampicin MIC values between isogenic strains as being biologically significant.

### Isolation of spontaneous mutants with increased MICs of corallopyronin A.

N. gonorrhoeae strains FA19, WHO Y, and WHO X were grown overnight on GCB agar as described above, and heavy growth from five plates were collected and resuspended in 5 mL GCB broth. Dilution plating onto GCB agar was performed to determine total CFU/mL, and 0.5-mL samples were plated onto GCB agar containing 2× or 4× the corallopyronin A MIC or 4× the rifampicin MIC. Plates were incubated as described above for 48 h before determining growth.

### Molecular techniques.

**PCR amplification of *mtrR* and *rpoB.*** The oligonucleotide primers used in this study for PCR amplification, which was performed as described previously (37), are listed in Table S2. For PCR amplification of *mtrR* oligonucleotide primers KH9#3 and CEL1 (Table S2A) were used, and the product was sequenced using CEL1. For amplification of *rpoB*, two pairs of oligonucleotide primers were employed: (i) rpoB1 and rpoB2 with the product sequenced using rpoB1, and (ii) rpoB3 and rpoB4 with the product sequenced using rpoB3, rpoB4, rpoB5, and rpoB6 (Table S2A).

**Cloning and transformation.** The *mtrR27* allele was PCR-amplified from the identified spontaneous mutant using oligonucleotide primers KH9#3 and CEL1, purified, and used to transform strain FA19 as described (39). Transformants were selected for increased resistance to erythromycin (0.5 μg/mL), and resistant colonies were counter-screened for increased resistance to corallopyronin A as described above. The presence of the *mtrR27* allele in transformants was confirmed by DNA sequencing of PCR products from three transformants as described above.

**qRT-PCR.** N. gonorrhoeae cultures containing wild-type or mutant *mtrR* alleles were grown in GC broth at 37°C with agitation to late exponential phase before being used for RNA purification and qRT-PCR as described previously (37, 39). Relative expression values were calculated as: 2(*C_T_* reference − *C_T_* target), where *C_T_* is the fractional threshold cycle. The levels of *recA* mRNA were used as internal references. The relative expression of each sample was normalized to the average relative expression of the wild type to obtain the normalized expression ratios. The following primer pairs were used to quantify relative mRNA levels: mtrR-qRT-F/mtrR-qRT-R for *mtrR*, mtrC-qRT-F/mtrC-qRT-R for *mtrC*, rpoH-qRT-F/rpoH-qRT-R for *rpoH*, rmpM-qRT-F/rmpM-qRT-R for *rmpM*, and recAqFw/recAqRv for *recA*.

10.1128/msphere.00270-22.2 TABLE S2 Table of oligonucleotide primers. Download Table S2, DOCX file, 0.01 MB. Copyright © 2022 Edwards et al. 2022 Edwards et al. https://creativecommons.org/licenses/by/4.0/ This content is distributed under the terms of the Creative Commons Attribution 4.0 International license.

**Database of** **N. gonorrhoeae** **proteins from public genome projects.** On 8 November 2021, we used the ncbi-genome-download tool (version 0.3.0; https://github.com/kblin/ncbi-genome-download) to download a database of proteins from N. gonorrhoeae genomes using the following command: ncbi-genome-download –species-taxids 485 –formats ‘protein-fasta’ bacteria. The proteomes of 731 genomes were downloaded. We searched this database using BLASTP (50) for orthologs of strain FA1090 MtrR with at least 90% sequence identity. All 731 strains had an MtrR ortholog, and these represented 35 individual NCBI protein IDs, equivalent to amino acid sequence alleles. To search for the allele, we hand-edited a file with residues 16 to 45 of FA1090 MtrR and created a variant with a G27R substitution. In all cases, the wild-type peptide had a higher BLAST score and percent identity to MtrR proteins than did the G27R variant, proving that this substitution was not present in the database.

**Whole-genome sequencing.** Genomic DNA was prepared from FA19 and its spontaneous mutant which showed a 4× increase in corallopyronin A MIC (see the text above), as described previously (34, 37). The DNA was sequenced at the WHO Collaborating Centre for Gonorrhoea and Other STIs (Örebro, Sweden). A fully automated Microlab Star (Hamilton, Reno, NV) was used to prepare sequencing libraries, quality control of the libraries, and subsequent library normalization with the Illumina DNA Prep (Illumina, Inc., San Diego, CA) and Qubit reagents (Thermo Fisher Scientific, Waltham, MA), respectively. WGS was performed on the Nextseq 550 system (Illumina) with read lengths of 149 bp.

**Data analysis.** Raw data were converted; fastq files were obtained and demultiplexed using bcl2fastq (v2.20.0.422) with the ‘–no-lane-splitting’ option. Fastq files from the isolates were quality controlled, trimmed, and mapped to the annotated reference sequence of N. gonorrhoeae FA19 (NCBI accession ID CP012026.1; 2,232,367 bp); variants were called, and functional consequences were identified using a customized workflow in CLC Genomics Workbench (v22.0.1).

### *Ex vivo* testing of corallopyronin A activity.

**Cell culture.** De-identified cervical tissues were obtained from the Cooperative Human Tissue Network (Columbus, OH) and used to procure primary human cervical epithelial (i.e., Pex) cells as described previously (51). In brief, this process involves the outgrowth of epithelial cells from dissected cervical tissue. Tissue explants and Pex cells were maintained using defined keratinocyte serum-free medium (dk-SFM; Gibco, Grand Island, NY). The N. gonorrhoeae strains used included the laboratory strain, 1291 (52), and a panel of MDR and XDR strains (WHO M, WHO X, WHO Y, and WHO Z) (30). Strain 1291 is a male urethral isolate that has been retained at a low passage number.

**Infection and treatment assays.** To mimic the cervical microenvironment, Pex cells and bacteria were cultured overnight in a tri-gas incubator under microaerobic (3% O_2_) conditions before their use in infection studies. Infection studies were then performed as previously described using a multiplicity of infection (MOI) of 100 (9). In brief, Pex cells were challenged with N. gonorrhoeae for 90 min to establish infection, after which the infection medium was replaced with fresh medium containing either 0.1% DMSO (vehicle control), corallopyronin A (0.25 μg/mL to 8 μg/mL), or ceftriaxone (0.5 μg/mL, positive control). Infections then proceeded for an additional 24 or 48 h (37°C, 3% O_2_), after which Pex cell monolayers were subsequently rinsed and lysed. Serial dilutions of the Pex cell lysates were plated, and viable gonococci were enumerated by counting CFU after a 48-h incubation (37°C, 5% CO_2_). The percentage of N. gonorrhoeae that survived corallopyronin A or ceftriaxone treatment was determined as a function of bacteria that survived DMSO treatment (set to 100%). All assays were performed in triplicate on 3 separate occasions. A nonparametric analysis of variance was used to determine the statistical significance of bacterial survival (GraphPad v8.2.0 for MacOs; GraphPad Software, San Diego, CA).

**Pex cell viability assays.** To determine whether corallopyronin A exerted a cytotoxic effect toward uninfected Pex cells, cells were incubated for 24 or 48 h with 0.1% DMSO (vehicle control), corallopyronin A (4, 8, or 16 μg/mL), or 0.5 μg/mL ceftriaxone, or they were left untreated. Pex cell viability was determined fluorometrically using a Cell Viability Assay kit (Fluorometric-Blue), according to the manufacturer’s instructions (Abcam, Waltham, MA). Assays were performed in duplicate on 3 separate occasions. Data were adjusted for background, after which fluorescence recorded for each condition was normalized to fluorescence recorded for untreated Pex cells. A Student’s *t* test (GraphPad) was used to determine the statistical significance of data obtained.

**Analyses of** **N. gonorrhoeae** **biofilms.** The medium used for Pex cell and abiotic biofilm growth comprised of a 1:1:2 mixture of Waymouth’s medium (Gibco, Grand Island, NY), Ham’s F12 medium (Gibco), and brain heart infusion broth (Becton, Dickinson and Co., Sparks, MD), respectively (WF12-BHI). To determine the effect of corallopyronin A on N. gonorrhoeae biofilms on an abiotic surface, 5 × 10^5^ of each gonococcal strain (WHO M, WHO X, WHO Y, or WHO Z) was added to select wells of a 24-well microtiter plate. Bacterial density was then read (absorbance at 600 nm) using a Synergy HT multimode plate reader (BioTek Instruments, Winooski, VT) to ensure that equal numbers of bacteria were present in each well. To initiate biofilm formation on host cells, Pex cells in 24-well plates were left uninfected or infected with gonococci at an MOI of 100, as outlined above. Microtiter plates (abiotic and biotic) were incubated at 37°C for 4 days to allow static biofilm formation. The medium was then replaced with either unsupplemented WF12-BHI or WF12-BHI supplemented with either 0.1% DMSO, 4 or 8 μg/mL corallopyronin A, or 0.5 μg/mL ceftriaxone. Plates were incubated for an additional 24 or 48 h, after which planktonic bacteria were removed and the wells were gently washed 3 times with phosphate-buffered saline. The final wash solution was discarded, and plates were incubated for 40 min at 75°C to dry. Pex cells and biofilms were stained for 15 min using 0.1% CV and extensively rinsed with double-distilled water (ddH_2_O) until it ran clear. Plates were dried overnight before the CV-stained biofilms were solubilized in 30% acetic acid. An aliquot of the CV-acetic acid solution was diluted 1:4 in ddH_2_O, and absorbance, indicative of biofilm biomass, was read at 590 nm. For both abiotic and biotic biofilm growth, data were adjusted for background. For Pex cell infection assays, data were also adjusted for uninfected Pex cell CV-staining; data shown represent N. gonorrhoeae biofilm biomass only. Each assay was performed using triplicate wells and on 3 separate occasions. The statistical significance of data obtained was determined using a Student’s *t* test (GraphPad).

**Scanning electron and confocal microscopy**. The microscopes used in these experiments were located at The Research Institute at Nationwide Children’s Hospital (Columbus, OH). For SEM analysis of N. gonorrhoeae biofilms, Pex cells were passed to placental collagen-coated coverslips and allowed to grow to confluence before infection with the noted strains, as described above. Biofilms were allowed to form for 4 days before processing, essentially, as previously described (53). In brief, cells were processed with 1% osmium tetroxide and dehydrated using a graded ethanol series, followed by a final incubation in hexamethyl-disilazane (HMDS; Polysciences, Inc., Warrington, PA). Coverslips were sputter coated with gold-palladium and viewed using a Hitachi S-4800 field-emission scanning electron microscope.

For confocal microscopy of gonococcal biofilms on human cells, Pex cells were passed to collagen-coated coverslips, as above. Upon confluence, Pex cells were infected with the noted N. gonorrhoeae strains, also as described above. Alternatively, for analysis of biofilms on an abiotic surface, 10^5^ of each strain was inoculated onto tissue culture-treated chamber slides (Cellvis, Mountain View, CA). Cells and slides were incubated (37°C, 5% CO_2_) for 48 h to allow biofilm formation. The infection/inoculation medium was then replaced with fresh medium containing 8 μg/mL corallopyronin A, 0.5 μg/mL ceftriaxone, 0.1% DMSO (vehicle control), or nothing and incubated for an additional 48 h. Biofilms were stained using the Live/Dead BacLight Bacterial Viability kit (Thermo Fisher), according to the manufacturer’s instructions. Biofilms on Pex cells were washed before staining; however, to prevent biofilm disruption, biofilms on chamber slides were not washed before staining. Analyses of the effects of corallopyronin A, ceftriaxone, and DMSO treatment on established N. gonorrhoeae biofilms was achieved using a Zeiss LSM800 (Carl Zeiss Microscopy, LLC., White Plains, NY) laser scanning confocal microscope with an Airyscan module (Zeiss). Images were captured using diode laser wavelengths of 488 nm (live bacteria) and 561 nm (dead bacteria) and collected as 1-μm Z-stack slices under 63× oil emersion. Z-plane images in which Pex cells were visible underneath gonococci were excluded from the construction of 3D N. gonorrhoeae biofilm images to avoid potentially confounding fluorescence resulting from host cell staining with Syto-9 and/or propidium iodide (i.e., with the use of the Live/Dead BacLight Bacterial Viability kit). Thereby, 3D images shown represent fluorescence captured as a result of N. gonorrhoeae biomass only. Biofilm architecture, total biomass (μm^3^/μm^2^), and the ration of live to dead bacteria were determined using ImageJ and Comstat (54) software.
