# Supplementary material for: Potent In Vitro and Ex Vivo Anti-Gonococcal Activity of the RpoB Inhibitor Corallopyronin A
Source: mSphere. 2022 Sep 12;7(5):e00362-22. doi: 10.1128/msphere.00362-22 (PMC9599356; doi:10.1128/msphere.00362-22)
Supplement: TABLE S1 [file msphere.00362-22-s0001.docx]

**Table S1. Knockout (KO) of MtrCDE in Ng strain FA19 increases gonococcal susceptibility to CorA**

Strain^a^ CorA MIC (µg/ml)

WT 0.125

FarAB-MtrE KO 0.125

MacAB-MtrE KO 0.125

NorM KO 0.125

MtrF KO 0.125

MtrCDE KO 0.03

^a^ All efflux pump KO strains are in the FA19 genetic background. This panel of efflux pump KO strains has been previously reported (2).
