# Supplementary material for: Potent In Vitro and Ex Vivo Anti-Gonococcal Activity of the RpoB Inhibitor Corallopyronin A
Source: mSphere. 2022 Sep 12;7(5):e00362-22. doi: 10.1128/msphere.00362-22 (PMC9599356; doi:10.1128/msphere.00362-22)
Supplement: FIG S1 [file msphere.00362-22-s0003.docx]

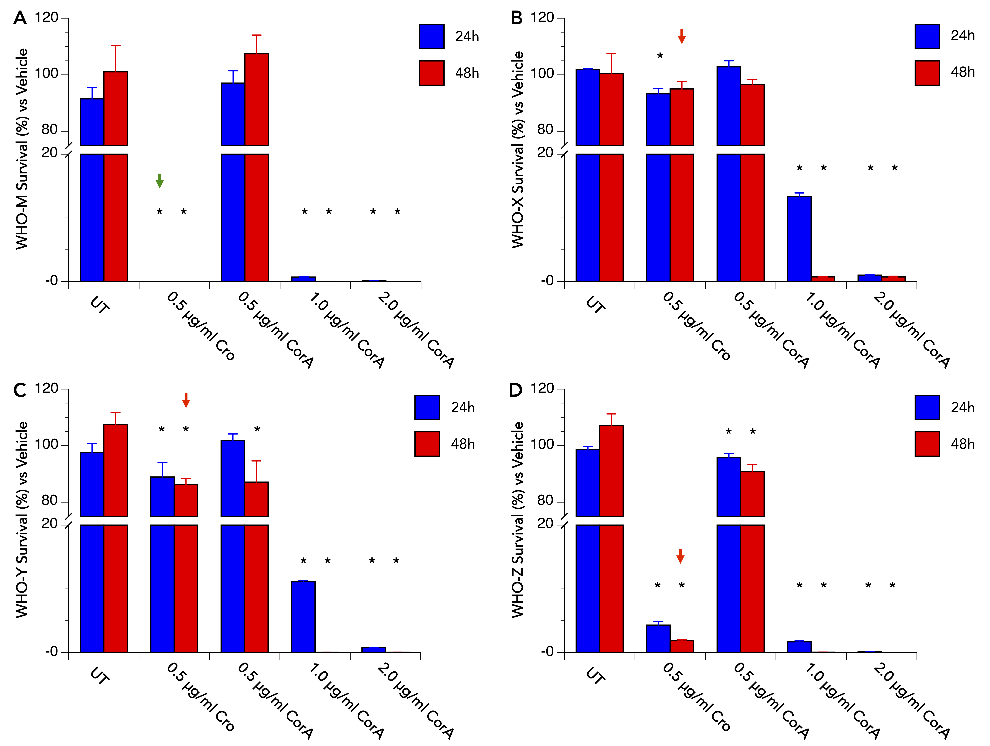


**Figure S1. Low dose corallopyroninA (CorA) treatment reduces the number of viable MDR or XDR *N. gonorrhoeae* during cervical cell infection.** The ability of strains WHO M (A), WHO X (B), WHO Y (C), and WHO Z (D) to survive a dose range (x-axis) of CorA treatment during Pex cell infection was examined, as described in the text. Values shown are the mean (variance) of the percentage of viable gonococci recovered at 24h (blue bars) or 48h (red bars) from untreated (UT) Pex cell infections or post-CorA or – ceftriaxone (Cro) treatment when compared to the number of viable bacteria recovered following treatment with the DMSO vehicle control at the same time point. Data were obtained from three trials performed in triplicate. A) The green arrow indicates that no viable gonococci were recovered from WHO M-infected Pex cells by 48h post-Cro treatment. B-D) The red arrows highlight that a 48h, 0.5 µg/ml treatment of Cro was not effective against strains WHO X, Y, and Z. * *p* ≤ 0.0196 versus vehicle.
