# Supplementary material for: Potent In Vitro and Ex Vivo Anti-Gonococcal Activity of the RpoB Inhibitor Corallopyronin A
Source: mSphere. 2022 Sep 12;7(5):e00362-22. doi: 10.1128/msphere.00362-22 (PMC9599356; doi:10.1128/msphere.00362-22)
Supplement: FIG S2 [file msphere.00362-22-s0004.docx]

**Figure S2. Corallopyronin A (CorA) is effective in disrupting an established *N*. *gonorrhoeae* biofilm on an abiotic surface.** The ability of CorA to disrupt a 4-day biofilm, formed on plastic multi-well dishes following inoculation with *Neisseria gonorrhoeae* strains WHO M (A), WHO X (B), WHO Y (C), and WHO Z (D), was determined following a 24h (blue bars) or 48h (red bars) treatment with CorA or ceftriaxone (Cro), as noted on the x-axis. Biofilm biomass was assessed by crystal violet retention, as described in the text. Data were adjusted for background and represent the mean (variance) bacterial biomass from triplicate wells from 3 experiments performed on separate occasions. * *p* ≤ 0.0045 versus vehicle control.
