# Supplementary material for: Potent In Vitro and Ex Vivo Anti-Gonococcal Activity of the RpoB Inhibitor Corallopyronin A
Source: mSphere. 2022 Sep 12;7(5):e00362-22. doi: 10.1128/msphere.00362-22 (PMC9599356; doi:10.1128/msphere.00362-22)
Supplement: FIG S3 [file msphere.00362-22-s0005.pdf]

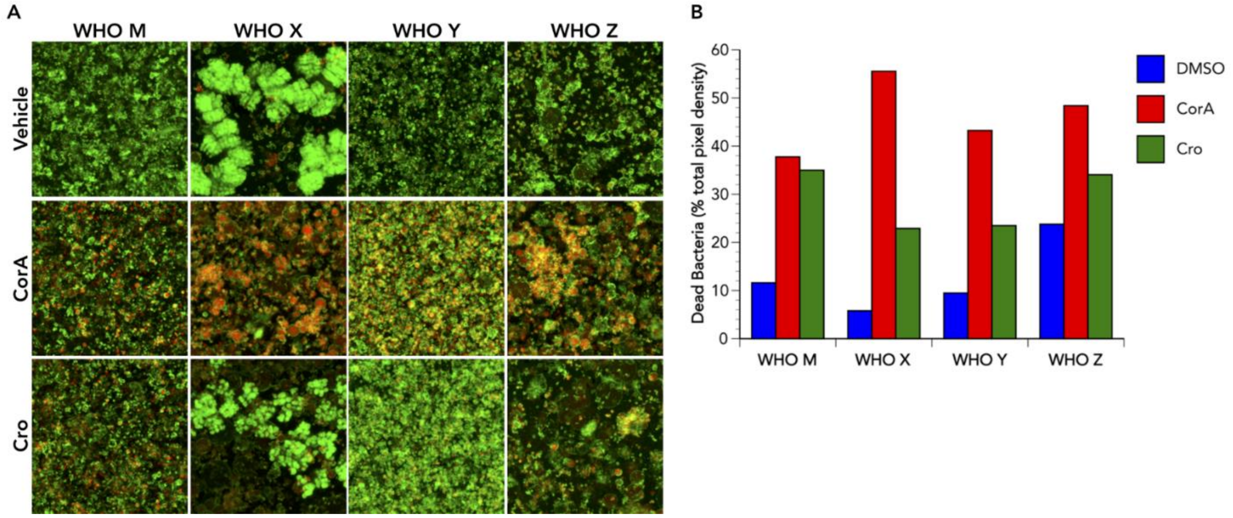

Figure S3. Confocal microscopic analyses of the effect of corallopyronin A (CorA) on *Neisseria gonorrhoeae* viability within abiotic biofilms. Tissue culture-treated chamber slides were inoculated with the MDR strains WHO M, WHO X, WHO Y, or WHO Z, as noted. Biofilms were stained using the BacLight Bacterial Viability Kit to distinguish viable (green) from dead (red-orange-yellow) bacteria, and analyzed as described within the text. A) Representative stacked-z images of 2-day Ng biofilms that were treated for an addition 48h with 0.1% DMSO (vehicle control), 8 µg/ml CorA, or 0.5 µg/ml ceftriaxone (Cro), as noted, are shown. x63 magnification with oil emersion. B) The percentage of dead bacteria are shown as a function of total biofilm biomass. Data shown were obtained through Comstat (3) analysis of the images shown in panel A.
