# Supplementary material for: Potent In Vitro and Ex Vivo Anti-Gonococcal Activity of the RpoB Inhibitor Corallopyronin A
Source: mSphere. 2022 Sep 12;7(5):e00362-22. doi: 10.1128/msphere.00362-22 (PMC9599356; doi:10.1128/msphere.00362-22)
Supplement: TABLE S2 [file msphere.00362-22-s0002.docx]

**Table S2. Oligonucleotide primers used in this study**

1. **Primers for PCR amplification for DNA sequencing**

| Primer Name |  | Sequence (5’ to 3’) |
| --- | --- | --- |
| RpoB1 |  | ATATCAGATTGATGCGTACCGTT |
| RpoB2 |  | CGTACTCGACGGTTGCCCAA |
| RpoB3 |  | GGCGGTTATGTCCTGAGCG |
| RpoB4 |  | GGCGGATTTTCGATATGGATAA |
| RpoB5 |  | CGCGCATTGATGGGTGCCA |
| RpoB6 |  | CGCCGATACCTTTTGCCGC |
| KH9#3 |  | GACGACAGTGCCAATGCAACG |
| CEL1 |  | GACAATGTTCATGCGATGATAGG |

1. **Primers for qRT-PCR**

| **Primer Name** | **Sequence (5’ to 3’)** |
| --- | --- |
| mtrR_qRT_F | CTTGTTTGACGCGTTGTTCCA |
| mtrR_qRT_R | GTGGATGTCGTTGCTTTGCA |
| mtrC_qRT_F | CGGATTTGGCGCGTTACAAA |
| mtrC_qRT_R | TAATGCGCGAACGGTTCAGA |
| rpoH_qRT_F | AACGGCAGCCTCGAACAATA |
| rpoH_qRT_R | GGTGGGACAGGATGAGTTGTT |
| rmpM_qRT_F | AAGCCAAGGTCGCGTAGAAT |
| rmpM_qRT_R | GGCGCGCAATGAATCCTTAT |
| recAqFw | AACCTCGAAGTCATTTCCACCGG |
| recAqRv | TCTGGCATTGGGCGACGGCTTC |
